# Reconstructed Human Epidermis: An Alternative Approach for In Vitro Bioequivalence Testing of Topical Products

**DOI:** 10.3390/pharmaceutics14081554

**Published:** 2022-07-26

**Authors:** Ana Sofia Agonia, Ana Palmeira-de-Oliveira, Catarina Cardoso, Cátia Augusto, Christian Pellevoisin, Christelle Videau, Ricardo Jorge Dinis-Oliveira, Rita Palmeira-de-Oliveira

**Affiliations:** 1 Labfit—HPRD Health Products Research and Development, Lda, Edifício UBIMEDICAL Estrada Municipal 506, 6200-284 Covilhã, Portugal; sofia.ferreira@labfit.pt (A.S.A.); apo@labfit.pt (A.P.-d.-O.); 2 CICS-UBI—Health Sciences Research Center, University of Beira Interior, Av. Infante D. Henrique, 6200-358 Covilhã, Portugal; 3 Faculty of Health Sciences, University of Beira Interior, Av. Infante D. Henrique, 6200-358 Covilhã, Portugal; 4 Laboratórios Basi, Mortágua, Portugal, Parque Industrial Manuel Lourenço Ferreira, Lote 15, 3450-232 Mortágua, Portugal; catarina.cardoso@basi.pt (C.C.); catia.augusto@basi.pt (C.A.); 5 EPISKIN, EPISKIN 4, Rue Alexander Fleming, 69366 Lyon, France; cpellevoisin@urbilateria.com (C.P.); cvideau@episkin.com (C.V.); 6 TOXRUN—Toxicology Research Unit, University Institute of Health Sciences (IUCS), Advanced Polytechnic and University Cooperative (CESPU), CRL, 4585-116 Gandra, Portugal; ricardinis@med.up.pt; 7 Department of Public Health and Forensic Sciences, and Medical Education, Faculty of Medicine, University of Porto, 4099-002 Porto, Portugal; 8 UCIBIO-REQUIMTE, Laboratory of Toxicology, Department of Biological Sciences, Faculty of Pharmacy, University of Porto, 4099-002 Porto, Portugal; 9 MTG Research and Development Lab, Rua Professor Joaquim Bastos, 4200-604 Porto, Portugal

**Keywords:** bioequivalence in vitro, permeation tests, reconstructed human epidermis, human skin, topical products

## Abstract

The use of in vitro human skin permeation tests is of value when addressing the quality and equivalence of topical drug products in Europe and the US. Human skin is the membrane of choice for these studies. The use of human skin as a membrane is hindered by limited access, high variability of results, and limited applicability for drugs with low skin permeability. Reconstructed human epidermis (RhE) models are validated as skin surrogates for safety tests and have been explored for percutaneous absorption testing. Clotrimazole poorly permeates human skin and is widely available for topical treatments. In this study, clotrimazole creams were used to test the ability of RhE to be used as biological membrane for bioequivalence testing, based on the Draft Guideline on Quality and Equivalence of Topical Products (CHMP/QWP/708282/2018) using a discriminative and modified in vitro permeation test (IVPT). To fulfill the validation of a discriminatory method, Canesten^®^ 10 mg/g cream was compared with a test product with the same drug strength, along with two “negative controls” dosed at a 50% and 200% drug strength. Products were compared in finite dose conditions, regarding maximal flux (J_max_) and the total amount of drug permeated (*A*_*total*_). The results showed the discriminatory power of the method among the three drug strengths with no interference of the placebo formulation. The study design and validation complied with the requirements established in the guideline for a valid IVPT. This new test system allowed for the equivalence comparison between test and comparator product. Higher permeability of the RhE compared to human skin could be observed. This arose as a strength of the model for this modified IVPT bioequivalence testing, since comparing permeation profiles among products is envisaged instead of drawing absolute conclusions on skin permeation extent. These results may support the acceptance of RhE as biological membranes for modified IVPT in bioequivalence testing of topical products.

## 1. Introduction

The bioequivalence of drug products has been typically assessed through clinical trials. For topical products, in particular, the use of in vitro drug release (IVRT) and in vitro drug permeation testing (IVPT) has been gathering increasing attention by both the US FDA [1] and, more recently, in Europe [2] for regulatory acceptance in specific situations. While for transdermal patches, these studies have been previously suggested for bioequivalence studies [2], a recently published “Draft Guideline on Quality and Equivalence of Topical Products” specifically addresses the use of this test also for topical drug products [3]. IVPT aims to establish the characteristic permeation profile of drug products (test and reference) comparing relevant parameters among them to assess their equivalence. IVPT is just one of the studies that support marketing authorization application of a new topical product. For an independent approach, equivalence demonstration should consider quality, efficacy, and safety, which together claim a therapeutical equivalence if the method of administration and the risk of inequivalence to the patient are minimal [3].

The Draft Guideline, CHMP/QWP/708282/2018 [3], issued by the Committee for Medicinal Products for Human Use (CHMP) by the European Medicines Agency (EMA), includes support for the development and consideration on equivalence with respect to efficacy of topical products. One of the studies provided is related to the in vitro human skin permeation test, suitable when the active substance diffuses through the skin to enable quantification in the receptor cell. A discriminative IVPT must be performed in a parallel study design with a test and comparator product. The study design in these types of studies pursue the characterization of the permeation profile of the active pharmaceutical ingredient (API) through biological membranes [4,5]. The experimental conditions, such as the skin membrane, the receptor medium, the number of sampling timepoints, and the dosing amount, should be justified. Method validation should include evidence of the appropriateness of the test conditions by using batches with different quality attributes, such as a negative control formulation with different strengths from the test product to assess the discriminatory power of the developed method.

The gold standard membrane for IVPT is the ex vivo adult human skin, obtained from the mammary or abdominal region of female donors. Several skin preparations can be used, and the selection must be justified. After the skin preparation step, the biological membrane is mounted on the top of the receptor part of the diffusion cell. These vertical diffusion cells are composed of a donor chamber and a receptor chamber that, as the name implies will embrace the receptor solution where the API from the topical product will permeate. In between these two chambers, the ex vivo human skin is placed on top of the receptor chamber, and the system is closed by placing the donor chamber on top of the membrane. Full-thickness and dermatomed skin may be used to include different layers of the skin, but performing the in vitro permeation testing with epidermis is often preferred, since stratum corneum (SC) is the main barrier for skin permeation of drugs [4]. Stratum corneum is usually prepared by immersing full-thickness skin in hot water for brief seconds. This technique is also known as heat-separated epidermis (HSE) [4,6,7,8,9]. Other approaches to isolate skin layers include chemical or enzymatic separation, but those are rarely used techniques.

Even though there are guidelines regarding the study design for IVPT, an approved standard with a detailed protocol is not yet available. There are only a few considerations to pursue. In fact, it was revealed that the correlation between in vivo and in vitro largely depends on the protocols followed [10,11]. The authors also stressed the importance of a harmonized protocol to optimize and standardize the hypothesis of a good correlation [10,11].

The use of human skin for such tests is important for in vitro–in vivo correlation purposes. However, using this membrane may be highly challenging. The availability of human skin that complies with inclusion criteria (e.g., integrity, absence of tattoos, and stretch marks) is very limited, and even when accessible, the skin grafts are too small to allow for a replicate study design. Even when adequate researcher qualification is assured for the sake of reliable results, variability is very significant and inevitable, even between different sections of the same donor. Moreover, while a permeation profile is mandatory for statistical comparison of the test outputs, the method cannot be used for drugs that cannot permeate human skin. It is well known that a drug’s physical and chemical characteristics influence its ability to permeate the skin (e.g., the molecular weight should be up to 400–600 Da and a log P within 1–4) [12]. Indeed, the Draft Guideline on Quality of Bioequivalence of Topical Products states that this is not applicable to whenever “it is not possible to measure a quantifiable permeation kinetic or pharmacodynamic event e.g., due to limited diffusion or insensitive tests” [3]. 

Because of those experimental limitations regarding ex vivo human skin, substantial research work has been conducted on reconstructed human skin models [13,14,15]. In 2010, ECVAM DB-ALM released a method summary regarding the use of reconstructed skin models specifically for percutaneous absorption testing [16]. These models have been validated as test systems for in vitro safety testing since 2013, as described in the OECD test guidelines related to skin corrosion (TG431), skin irritation (TG439), and skin phototoxicity (TG432). The OECD guidance document to conduct skin absorption studies (2004) states that “reconstituted human skin models can be used if data from reference chemicals are consistent with those in the published literature” [4]. Although RhE models are not currently approved for skin absorption testing of chemicals for regulatory purposes, they have been used for this purpose in cosmetics, pharmaceuticals, and industrial chemicals, even if it is for research purposes [17,18,19,20,21].

Several commercially available skin models of RhE (EpiSkin^®^, SkinEthic^®^, EpiDerm^®^, LabSkin^®^, and Phenion^®^FT) have already been used for in vitro permeation testing of several molecules such as salicylic acid, hydrocortisone, clotrimazole, testosterone, caffeine, flufenamic acid, mannitol, ivermectin, benzoic acid, nocitene, digoxin, terpenes, and carfentanil [13,22,23,24,25,26]. In fact, those models went through a successful validation process regarding their applicability for in vitro permeation testing. Among the nine substances tested with different molecular weights and lipophilicity, SkinEthic^®^ showed to be more permeable than EPISKIN^®^ and EpiDerm^TM^, and all these RhE models showed to be more permeable than human and pig skin. These membranes are considered appropriate alternatives to human and pig skin for IVPT of substances when applied as aqueous solutions. Permeation through RhE, HSE, and pig skin resulted to be correlated with each other, except for the correlation with human skin uptake [13].

The use of RhE as an alternative membrane to excised human skin for percutaneous absorption and bioavailability screening is an appealing conjecture due to the higher batch-to-batch reproducibility, and its similarity to human skin [17,18,27], even if it is known that they present higher flux rates when compared to HSE. There is a consensus that reconstructed epidermis models are more permeable to test substances than excised human skin due to its incomplete barrier function [27], and a prediction model should, in fact, be the next step for development [13,28]. Regardless of all efforts in the introduction of RhE models to permeation studies [13,27,29,30], there are no current conclusions regarding the use of these models in bioequivalence studies for IVPT with the intent to compare test formulations with comparator ones and to establish a degree of equivalence.

Therefore, this study aimed to investigate the suitability of RhE models as test systems for bioequivalence studies of topical formulations, according to the requirements of study design and method validation described on the Draft Guideline on Quality and Equivalence of Topical Products (CHMP/QWP/708282/2018) [3]. The clotrimazole 1% test and reference topical products were selected for this study due to limited extension of skin permeation of this lipophilic drug [29] and to high variability encountered in in vitro human skin permeation testing with clotrimazole products [30,31,32,33].

## 2. Materials and Methods

### 2.1. Materials and Reagents

For the receptor solution preparation, PBS (free from Ca^2+^ and Mg^2+^, VWR), propan-1,2-diol (ACROS Organics^TM^), and sodium azide (VWR) were used. Methanol HPLC grade (Fischer Scientific, Thermo Fischer Scientifics^TM^, Waltham, MA, USA), KH_2_PO_4_ (VWR), and ortho-phosphoric acid (VWR) were used for the chromatographic analysis. Glass beads with 3–5 mm and syringe PTFE filters with a 0.45 µm pore diameter both from VWR. Biological membranes EPISJ13 U (EPISKIN^®^, Lyon, France) were purchased along with maintenance medium MAIN3.

### 2.2. Biological Membranes: Reconstructed Human Epidermis 

EPISKIN^®^ reconstructed human epidermis (RhE) tissue inserts were used as the biological system. This RhE model/supplier was not only validated, but it was also used for toxicological safety studies in the OECD test guidelines.

Each insert of RhE is made of type I collagen matrix representing dermis, surfaced with a type IV collagen film, upon which a stratified differentiated epidermis (i.e., basal layer, stratum spinous, stratum granular, and stratum corneum) derived from human keratinocytes is grown. These cells have 13 days of age with a 1.07 cm^2^ area. Each batch received is certified by a quality control test with histology testing (i.e., an HSE-stained paraffin section to observe the differentiated layers and count the number of layers), cell viability (at a 570 nm optical density by MTT test), and barrier function activity (exposure time inducing 50% viability using Triton X-100 1%).

The EMA guideline states that the number of skin donors for IVPT should be no fewer than 12, with at least two replicates per donor. In addition, the test and comparator products shall be tested using the same donors. This study was designed by following the Draft Guideline recommendations, i.e., using 12 tissue inserts per sample. Since batches of RhE are prepared based on pooled donor samples (and not individual donors), it is not expected that significant variability is encountered among different batches. However, five independent batches of tissue inserts were used on different days to report a total of 12 tissue inserts per formulation batch. Through this biological test system choice, influences of experimental variability from different sources can be considered (different batches and different test days). 

The inserts were shipped at room temperature in a 24-well plate filled with agarose-nutrient solution packed in a sterile plastic bag. Tissue manipulation was always performed inside the safety cabinet, under sterile conditions. On the day of the reception, tissues were visually inspected for any abnormality needed to be reported. Tissues were transposed to a new 12-well plate, where 2 mL of maintenance medium were previously pipetted. Tissue plates were placed in the incubator overnight (18–24 h) at 37 ± 1 °C, 5% CO_2_, and ≥90% relative humidity. After the overnight pre-incubation time, tissues were placed in a new 12-well plate with 2 mL of receptor solution, where the tissue and receptor medium stabilized for at least 30 min in the incubator. After this time, the receptor solution was replaced by 2 mL of fresh receptor solution, and the sample was topically applied on the top of the tissue insert (dosing). Tissues were kept in the incubator under mild agitation (250 rpm) throughout the study at 37 ± 1 °C, 5% CO_2_, and ≥90% relative humidity. At each defined timepoint, the plate was placed in the safety cabinet for collection of sampling timepoints into HPLC insert vials (See Supplementary Materials Figure S1).

### 2.3. Drug Products

Clotrimazole 1% creams were chosen as the study substances. The comparator of the bioequivalence test was defined as Canesten Antifungal Cream^®^ 10 mg/g cream, and the test product as clotrimazole 10 mg/g cream prepared by Laboratórios Basi (Mortágua, Portugal). Quality equivalence among these products was assessed previously through IVPT. Two negative controls (changed drug concentration) were selected to assess the discriminatory power of the method. Both were prepared by Laboratórios Basi, specifically the 0.5% and 2% clotrimazole creams, corresponding to 50% and 200% drug strength, with the same excipient composition of the test product. A placebo formulation with the same excipient composition and without API was also prepared using the same method by Laboratórios Basi. The qualitative composition and drug strength of the study samples are presented in Table S1 (Supplementary Materials). All tests in the bioequivalence study (after the pilot study) were performed with blinded products, except for the 50% test formulation (D, Table S1). This 50% drug strength formulation was also included in the study, as an additional product, to better understand the sensitivity of the test system. Unblinding was only processed after all experimental data were obtained and statistical analysis performed for the results presentation.

### 2.4. IVPT Method Pilot Study

According to the EMA’s Draft Guideline, a pilot study comparing the test and comparator product is advisable to verify that the active substance permeates through the skin and that experimental conditions are optimized [3].

After performing the pilot study assay, conditions for IVPT should be studied and previously defined. The test conditions used in the pilot study are described in Table 1. Although the Draft Guideline recommends a temperature of 32 °C (skin temperature) at the surface of the tissue [3], previous experiments used 37 °C for permeation purposes [34,35,36,37,38]. In addition, the experiment was performed in incubators with standardized conditions for cell and tissue culture. While corresponding to a deviation from skin temperature, the same temperature was used for all tested formulations which enabled a direct comparison between them.

Information on all deviations from the IVPT standard testing throughout this study design are included in Table S2 together with justifications for such deviations.

### 2.5. IVPT Method Study Design

The study design was based on “Annex II—in vitro skin permeation studies” of the “Draft guideline on quality and equivalence of topical products” (CHMP/QWP/708282/2018) [3].

All experimental conditions defined in the Draft Guideline were justified in the study design using the RhE model instead of the standardized ex vivo human skin.

Table S4 (Supplementary Materials) presents the detailed study design which includes the performed assays and the formulation designated for each RhE EPISKIN^®^ tissue insert. Each assay number translates into a different EPISKIN^®^ batch, totaling five independent batches. The study design assured that all products were tested in parallel in every assay. One tissue insert per batch was used for dosing the placebo formulation to guarantee that neither the tissue nor the formulation interfered in the quantification method. The membrane integrity evaluation was also considered.

Test conditions used for the bioequivalence evaluation and the method validation are described in Table 1.

Further experimental details and justification of the parameter selections are given in the following items.

#### 2.5.1. Dose and Sample Application

According to the Draft Guideline [3], the topical product was applied on the skin’s surface considering a finite dose methodology (2–15 mg/cm^2^) based on the summary of product characteristics (SmPC) to simulate conditions of use and to demonstrate the depletion of the drug along with the assay timeframe. The donor compartment should be unoccluded unless otherwise specified in the SmPC. According to the SmPC of the comparator product (i.e., Canesten^®^), the cream shall be applied as a thin layer approximately half a centimeter in length, two to three times per day. This weight was superior to 15 mg/cm^2^. Thus, the finite dose of this study was defined, considering all available information, as 15 mg/cm^2^. 

The amount of formulation to apply was calculated by weighting it in a tracing paper and through mass difference before and after application. Homogeneous spreading was guaranteed during application, with the aid of a sterile loop. See Section 2.2 for tissue protocol before and after dosing.

#### 2.5.2. Receptor Solution 

It must be assured that the solubility of the drug in the receptor solution will not be a limiting parameter for drug permeation, allowing for sink conditions to be maintained throughout the experiment. It was assumed that sink conditions were met if the maximum concentration of the drug achieved in the experiments did not exceed 10–30% of its maximum solubility in that receptor solution [3].

A physiological solution such as PBS pH 7.4 is preferred in permeation studies. The addition of a surfactant or other additive may be needed to comply with solubility requirements whenever poorly aqueous soluble drugs are studied. Since clotrimazole is poorly soluble in water, as stated by European Pharmacopoeia 10, the use of such an additive in the receptor medium was envisaged.

In preliminary studies, several mixtures were tested regarding the solubility of clotrimazole mixing a PBS solution with several different additives at different proportions (see Supplementary Materials Table S3). The relative solubility of clotrimazole in PBS 7.4: propan-1,2-diol (60:40, %*v*/*v*) was 495.0 µg/mL, demonstrating it to be an appropriate choice when compared to the other solubility solutions tested. Propan-1,2-diol is a known solvent and permeation enhancer that acts also by interacting with keratin [39,40,41,42]. This could be a limitation for the experimental setup. Still, the receptor solution was placed on the bottom of the tissue insert, meaning that it will not come into direct contact with the stratum corneum and, therefore, is not expected to directly enhance the lipophilic drugs’ permeation through the reported mechanisms. The tissue insert was stratified from the bottom to the top by a supportive type I collagen matrix representing the dermis, surfaced with a type IV collagen film, upon which there was a stratified differentiated epidermis with stratum spinosum, stratum granulosum and, on the top, stratum corneum. The latter was the top layer of the reconstructed human epidermis and the furthest from the receptor solution. The receptor solution does not contact directly with the top layer of the skin insert. This solvent was used mainly because of its capability to enhance clotrimazole solubilization in the receptor medium, assuring sink conditions throughout the in vitro permeation test. Still, measurement of tissue integrity is essential to mitigate the risk of tissue damage due to the high amount of solvent used in the receptor solution.

Sodium azide was added as a preservative to the receptor solution in a 0.02% (*w*/*v*) concentration as an antimicrobial substance to avoid decomposition of the biological membrane [3].

#### 2.5.3. Sampling Timepoints and API Quantification

The sampling timepoints for the study were defined in the pilot study. At each timepoint, 200 µL of receptor solution was collected and replaced by thermostatized and fresh receptor solution to maintain the 2 mL final volume in each well. Internal quality control (IQC) was performed before sample application to verify the absence of clotrimazole by HPLC.

Quantification of clotrimazole was performed through an HPLC-DAD method using a High-Pressure Liquid Chromatograph VWR/Hitachi Chromaster (models: CM 5160, 5310, and 5260) coupled with diode array detector (model: CM 5430). The chromatographic column used was an ACE Equivalence 5 C18 4.6 mm ID × 250 mm.

The mobile phase consisted of methanol and 25 mM K_2_HPO_4_ at pH 7.5 (75:25; %*v*/*v*) in the isocratic mode. A flow rate of 1.5 mL/min and a volume injection of 20 µL was programmed. The column temperature was set to 30 °C, and the autosampler was to refrigerate at 4 °C. Clotrimazole was detected at 210 nm with a run time of 10 min.

Stock solutions of 1 mg/mL were prepared in methanol. Working standard solutions and calibration curves were performed with appropriate diluent. Sampling timepoint quantification and tissue and donor chamber quantification for mass balance purposes used receptor medium and methanol, respectively. Method validation comprised the evaluation of several characteristics as described by the International Conference on Harmonization of Technical Requirements for Registration of Pharmaceuticals for Human Use (ICH). The guideline used for this validation was the ICH Tripartite Guideline “Validation of Analytical Procedures: Text and Methodology—Q2 (R1)” [43]. Several validation criteria were further considered according to the Reviewer Guidance “Validation of Chromatographic Methods” by the Center for Drug Evaluation and Research (CDER) [44].

#### 2.5.4. Mass Balance

Total recovery of clotrimazole was calculated as the mass balance according to the OECD [4] and EMA guidelines [2,3]. Clotrimazole’s recovery at the end of the experiments was calculated considering the mass of the formulation initially applied on the donor chamber, which allowed for the calculation of the 100% drug mass. The numerator of this quotient was translated as the sum of the final cumulative amount of clotrimazole (*A*_*total*_) that permeated the biological membrane into the receptor medium, the drug extracted from the formulation remaining in the donor chamber, and the drug extracted from the biological membrane at the end of the experiments. 

Extraction of the donor chamber was performed by removing the remaining formulation on top of the tissue with a loop and solubilizing it in 20 mL of methanol. Total removal of the remaining product was further achieved with a surgical gauze and extracted in the same solvent. After, the plastic insert was detached from the tissue and was submerged in 5 mL methanol. Following 15 min of sonication (no temperature) and verification that the solution was homogeneous, it was filtered with a 0.45 µm PTFE syringe filter. The membrane separated from the insert was put into a 2 mL methanol volume. Sonication and filtration were also performed like the plastic insert extraction process.

According to the EMA’s Draft Guideline, the mass balance should be within 100 ± 10%, corresponding to an assay quality criterium. Deviations from this range are also tolerable if justified, and Schäfer-Korting stated that a recovery of 100 ± 15% is only necessary to meet the validity criteria for mass balance calculations [13].

The mass balance was calculated according to Equation (1):

(1) $$\mathrm{mass}\;\mathrm{balance}=\frac{m_{\mathrm{donor}}+m_{\mathrm{permeated}\left(\mathrm{receptor}\right)\;}+m_{\mathrm{skin}}\;}{m_{\mathrm{applied}}\;}\times 100,$$

where $m_{\mathrm{applied}}\;$ is the total mass of drug applied on the skin’s surface, $m_{\mathrm{donor}}$ is the mass of drug recovered from the donor compartment at the end of the study, $m_{\mathrm{permeated}}\;$ is the cumulative mass that permeates the epidermis at the end of the assay, and $m_{\mathrm{skin}}\;$ is the mass of drug extracted from the membrane at the end of the assay.

#### 2.5.5. Biological Membrane Integrity

According to the Draft Guideline, there should be evidence to demonstrate that the skin did not undergo changes in the barrier function throughout the assay timeframe. This parameter should be assessed before applying the topical formulation and after the last sampling timepoint. 

Visual inspection was always performed for all tissues by means of a magnifying lens. TEER and/or TEWL measurements were performed in one or two tissues per assay as a representative batch measurement, mitigating the risk of damaging the tissue membrane during integrity checks.

In this study, two major monitorization methods to evaluate the in vitro skin integrity were used: trans-epithelial electrical resistance (TEER) and trans-epidermal water loss (TEWL). In addition to these methods, visual inspection was never discarded.

TEER was measured using a Millicell^®^ ERS-2 (Millipore^®^) equipment. The Millicell^®^ ERS-2 (Electrical Resistance System) is a meter and an electrode system designed to reliably measure TEER of epithelial cells in culture. An increase in TEER (Ωcm^2^) is an indication of cell monolayer health and confluence. Although it has been designed for monolayers, this method has been previously used for 3D and skin models [45,46,47,48].

TEER measurements on the day of the reception (D0), the day of sample application (D1), and/or the day that the assay ended (D3) were performed on the spare tissues ordered for assay#3 and assay#4 (13th tissue) that received the placebo formulation.

For TEWL measurements, condenser-chamber equipment was used (Aquaflux^TM^, model AF200, Biox Systems Ltd., London, UK) equipped with a stainless-steel tube insert (18 mm in length) coupled to a standard clamp cap. By this method, water vapor from the test surface (in this case, the tissue) is captured in the measurement chamber, which diffuses through the enclosed air and forms ice on the condenser. Higher TEWL values (g/m^2^h) represent less skin integrity, meaning that there is water through the skin. Further details on TEWL parameters programmed for the measurement are presented in Table S5.

A well-established TEWL value is not standardized by regulatory agencies for ex vivo human skin to consider that the membrane is intact. TEWL values of ex vivo human skin ranging from 10 and 20 mg/cm^2^/h [49,50,51,52] up to 30 mg/cm^2^/h have been reported [53]. Because the RhE model is a more permeable tissue [54,55], with a lower barrier function, it is expected that the TEWL value will be higher. In this study, it was considered that the TEWL reading at day D0 was the baseline and significant increases from this value indicated a reduction in the barrier function.

### 2.6. Presentation of Results: Permeation Profiles

The cumulative permeated amount of clotrimazole (*A*_*total*_) was calculated from Equation (2):

(2) $$A_{total}=\frac{Cn.V+\sum_{i=1}^{n-1}Ci.S\;}{A}$$

where Cn represents the concentration of clotrimazole determined at a certain sampling time; V is the volume of the receptor compartment; $\overset{n-1}{\underset{i=1}{\sum }}Ci$ the sum of the concentrations of clotrimazole determined from sampling time 1 to *n* – 1; S is the volume of the sampling aliquot (0.2 mL); A represents the surface area of skin exposed. 

The cumulative amount permeated per unit area as a function of time was further plotted as the rate of absorption (*J*, mass unit/cm^2^/h) versus time to allow for the determination of the maximal rate of absorption (J_max_) in the study’s timeframe. Flux values (*J*), for each timepoint, where calculated according to Equation (3):

(3) $$J=\frac{\;A_{total}}{t}$$

where $A_{total}$ is the cumulative permeated amount calculated from Equation (2) for the defined timepoint and t is the exposure time (in hours) at which the $A_{total}$ was calculated [56].

For a graphical representation of the permeation profiles of the products, the cumulative amounts of permeated drug per unit of the surface area of skin (µg/cm^2^) was plotted versus time. Calculations of flux for long intervals (0–48 h; 0–24 h; 26–48 h) were also performed through linear regression to allow for further analysis and discussion of results.

For each product under test, the means and coefficients of variation of *J* and *A*_*total*_ were calculated.

### 2.7. Presentation of Results: Bioequivalence Statistical Analysis

According to the Draft Guideline [3], the therapeutic equivalence study of a test and a comparator topical product should be assessed by calculating the 90% confidence interval (CI) for the ratio of the means of the test and comparator products. This interval should be within 80.00–125.00%, unless otherwise justified. Statistical analysis was performed using SPSS (version 25, IBM^®^ SPSS^®^ Statistics, Armonk, NY, USA) for the generalized linear model (GLM, univariate) analysis and further calculation of the CI 90% according to the following formula: lower limit for the ratio = 100 (1 + lower/RefLSM) and upper limit for the ratio = 100 (1 + upper/RefLSM), where “RefLSM” is the least square mean of the comparator formulation, “lower” is the lower confidence interval for the difference between the two formulations, and “upper” is the upper confidence interval for the difference between the two formulations.

## 3. Results

The use of IVPT to investigate the bioequivalence of topical drugs is a recently accepted strategy. However, it is limited to those drugs that can permeate human skin in a short time, allowing for a permeation profile to be drawn. Clotrimazole is a drug that is extremely challenging to test on human skin, not only because of its low permeation profile (due to the fact of its lipophilic chemical characteristic outside the range of optimal permeation characteristics) but also because of the high variability encountered when using these models. RhE is a more permeable model than human skin in absolute terms. This characteristic was used as a positive aspect in these models by allowing the comparison of an active substance characterized by its low in vivo permeation.

Intending validation and proof of bioequivalence through in vitro RhE permeation testing, four different clotrimazole formulations were studied using a physiological-like receptor solution, yet capable to solubilize this specific API without limiting skin conditions.

### 3.1. IVPT Method Pilot Study

**Figure 1:**
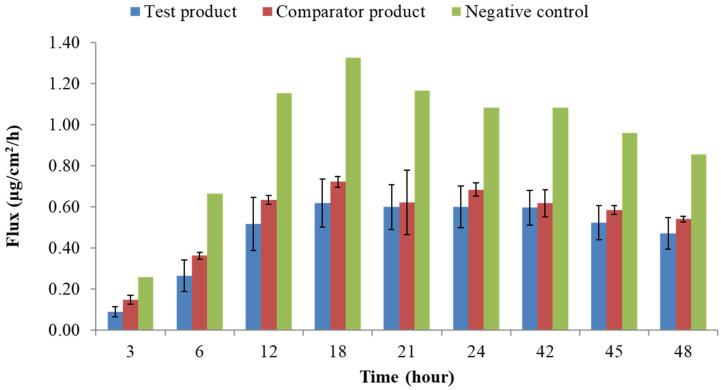
Flux (µg/cm^2^/h) obtained at each timepoint throughout the 48 h assay in the pilot study (*n* = 3 for the test and comparator products; *n* = 1 for the negative control; bars represent the mean with standard deviation).

**Figure 2:**
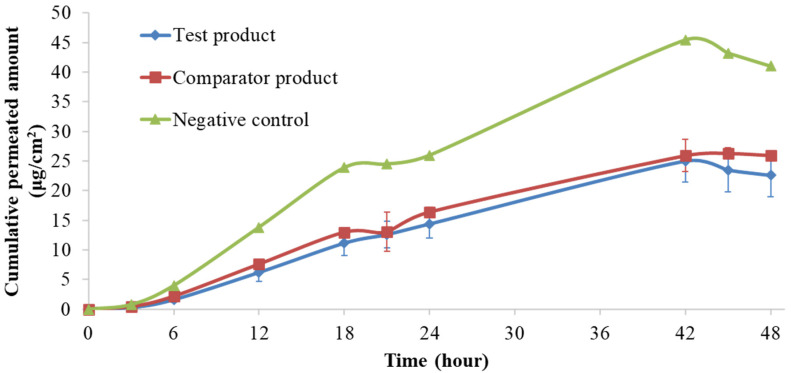
Cumulative permeated amount (µg/cm^2^) per hour obtained in the pilot study (*n* = 3 for the test and comparator products; *n* = 1 for the negative control; bars represent the mean).

The results obtained in this study are presented in Figure 1 and Figure 2. 

It was possible to readily unveil that the tested conditions enabled the permeation of clotrimazole. A placebo formulation and blank tissue did not interfere with the clotrimazole quantification method along the 48 h assay timeframe.

Concerning the API permeation profile, low coefficients of variation (<15%) were obtained, and the negative control formulation exhibited a higher flux when compared to test and comparator formulations. It was possible to disclose that the negative control formulation’s total cumulative amounts (41 µg/cm^2^) were approximately two-fold higher than those obtained for the test and comparator formulations (22.6 and 26.0 µg/cm^2^, respectively). The maximum fluxes were achieved between 12 and 18 h for all formulations.

At the 48 h timeframe, the flux reached a plateau (Figure 2), displaying the characteristic finite dose curve expected with the applied dose of 15 mg formulation, as the flux, J, between 26–48 h was less steep than the one observed between 0 and 24 h. 

The pilot study confirmed that the IVPT parameters (described in Table 1) were well defined, enabling the progression to bioequivalence testing with the study design specified in Table S4. The experimental conditions defined were adjusted in the number of sampling timepoints to better plot the permeation profile of clotrimazole. In addition, glass beads were used in the mass balance extraction to improve the extraction yield of clotrimazole (see Section 2.4 and Table 1).

### 3.2. IVPT Bioequivalence Study and Method Validation

The bioequivalence study was performed with two deviations related with insert 10 and 1 from assays #3 and #5, respectively: The extraction of the donor chamber for both inserts were spilled, and the mass balance parameter could not be calculated. For this reason, the results obtained with these inserts were rejected. The conceived randomization of the drug products and the effort to introduce all products, with the same n, into every assay, (corresponding to the best experimental practices) were modified in assay#5. Nevertheless, all drug products were studied in a total of 12 tissues each, so the EMA’s criterion was met for the minimum number of cells needed to evaluate bioequivalence.

### 3.3. Permeation Profile

The results obtained for the relevant permeation parameters are presented in Table 2.

**Figure 3:**
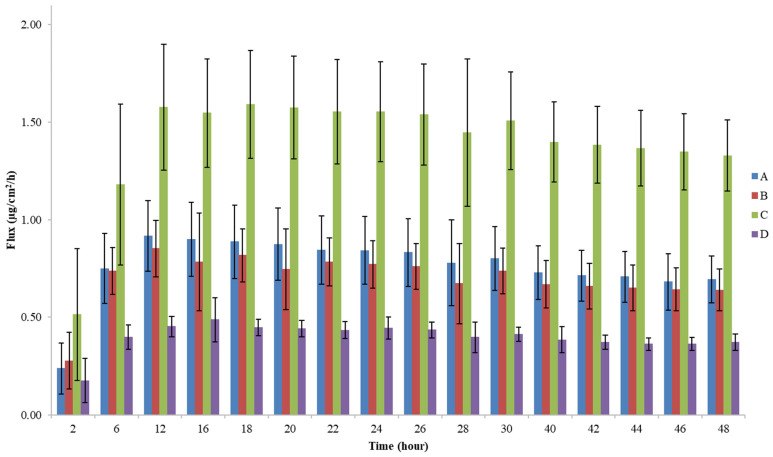
Flux (µg/cm^2^/h) obtained at each timepoint throughout the 48 h assay (*n* = 12) (A—clotrimazole 10 mg/g cream; B—Canesten Antifungal Cream 10 mg/g cream; C—clotrimazole 20 mg/g cream; D—clotrimazole 5 mg/g cream).

The plot of the rate of absorption (J, µg/cm^2^/h) as a function of time (48 h) to identify J_max_ is presented in Figure 3. 

**Figure 4:**
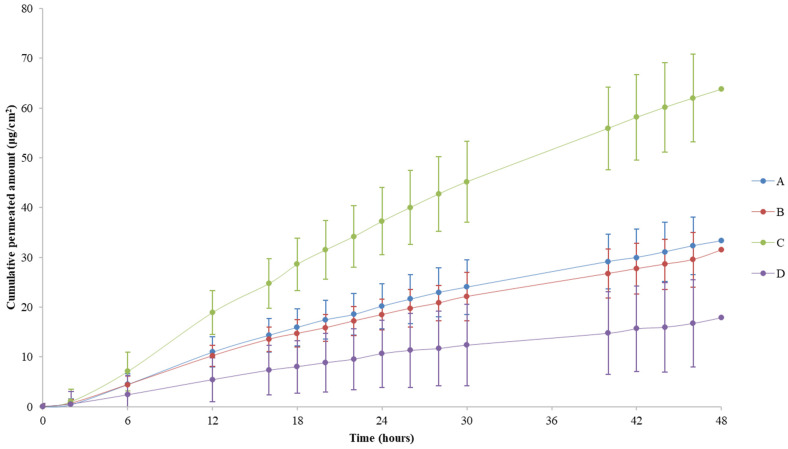
Cumulative permeated amount (µg/cm^2^) per hour obtained throughout the 48 h assay (lines represent the mean with SD, *n* = 12) (A—clotrimazole 10 mg/g cream; B—Canesten Antifungal Cream 10 mg/g cream; C—clotrimazole 20 mg/g cream; D—clotrimazole 5 mg/g cream).

Maximum flux (J_max_) in all formulations was achieved before 24 h, (t_max_ between a 12 and 20 h timeframe), suggesting a 24 h timeframe for comparison purposes. Nevertheless, by extending the assay up to 48 h, a more complete permeation profile was obtained. The mean cumulative amount (µg) of clotrimazole per surface area (cm^2^) that permeated the skin membrane over time (h) is also presented in Figure 4. Overall, a 24 h timeframe is preferred, as stated in the EMA’s Draft Guideline for permeation studies for mitigation of the risk of tissue damage over time [3].

Our results undoubtedly show that products that only differ in drug strength (A, C, and D formulations) presented different fluxes (J and J_max_) and different cumulative amounts of permeated clotrimazole (*A*_*total*_) (Figure 3 and Figure 4, Table 2). Furthermore, J_max_ and *A*_*total*_ were ordered in accordance with drug strengths as expected: formulation C with 2% clotrimazole (1.63 µg/cm^2^/h and 37.27 µg/cm^2^) > formulation A with 1% clotrimazole (0.94 µg/cm^2^/h and 20.23 µg/cm^2^) > formulation D with 0.5% (0.51 µg/cm^2^/h and 10.68 µg/cm^2^), 24 h results. Consequently, the method presented discriminatory power. 

When analyzing the variability of the results, using the coefficient of variation (in percentage), the maximum variation obtained for J_max_ calculation was 21.43% for the test 5 mg/g formulation.

Considering the biological nature of the study system, this coefficient of variation is acceptable. Indeed, much higher variations were observed in ex vivo experiments with human skin [57,58,59]. EMA’s Draft Guideline does not specify the acceptance criteria for this variation.

It is also possible to recognize that formulations A and B presented comparable permeation profiles over the 48 h timeframe (Figure 4 and Table 2) with t_max_ mean values around 15 h (in the timeframe 12–16 h) for both. Indeed, the results obtained closely overlap throughout the assay. Formulation C has a much higher permeation profile when compared to formulations A and B. The profile of the formulation with 50% of the test product strength (formulation D) was lower than the A and B formulations and, thus, lower than formulation C. Maximum fluxes were achieved after 12 h (Figure 3).

### 3.4. Biological Membranes Integrity

Visual inspection was always performed for all tissues by means of a magnifying lens, and no meaningful damages were observed. 

#### 3.4.1. TEER

TEER results are presented in Table 3.

On assay#3, TEER measurements intended to study possible variations throughout the testing days. Indeed, it was possible to verify a reduction in the TEER value after overnight incubation in the maintenance medium (from day D0 to day D1). We hypothesized that this could be due to negative effects of the receptor solution placed on top of the tissue while performing these measurements. Still, the difference between day D1 and day D3 was not significant. Thus, this confirms that neither the formulation nor the receptor solution placed below the insert affected the tissue integrity between sample application and the end of the testing. On the same assay (#3), TEER measurement was also performed on insert 11, which received the comparator formulation. The result did not differ from the one obtained in the placebo formulation (insert 13).

On assay#4, it was possible to further confirm that the decrease in TEER observed on assay#3 from day D0 to D1 was not due to the receptor solution placed on top of the insert for TEER reading on day D0, since it occurred also with PBS pH 7.4 and maintenance (culture) medium. Therefore, we concluded that there was no interference from the sample application (placebo versus comparator formulation). Also, the use of receptor solution at the top of the membrane did not negatively impact the measurement compared to PBS and maintenance medium. This intrinsic decrease in the TEWL of the EPISKIN^®^ insert membrane was probably due to loss of cellular viability in the absence of maintenance culture medium (from D1 to D3). Despite the TEER decrease compared to day D0, it did not translate into higher flux (Table 2 and Figure 3).

#### 3.4.2. TEWL

The obtained values were considered acceptable (Table 4).

On assay#1, TEWL readings were performed by handling the equipment, while on assay#2 readings were performed with the aid of a static stand with clamp and elevator. The latter reading method resulted in increased control over measurements.

A more evidenced increase in TEWL between day D1 (formulation application day) and D3 (final day) on assays #1, #4, and #5 (Table 4) was observed. This did not translate into a higher permeation rate when compared with the other tissues indicating that it did not affect the permeation of clotrimazole (data not shown). This increase may be related to the removal of the formulation from the top of the insert, which might impair tissue integrity. In fact, if membrane damage occurred at some point, it would be expected that the drug concentration in the receptor solution would increase significantly after 24 h of permeation. According to the results, a disproportional increase in the cumulative amount of drug permeated was not noticeable. Moreover, a finite dose-like plateau was observed in the respective cumulative permeated amount (µg/cm^2^) per hour, evidenced by flux calculations at each timeframe (Table 2). By analyzing the timeframes of the flux (µg/cm^2^/h), a decrease in the rate of permeation after the 24 h timeframe was perceived (between 26 and 48 h; Figure 3 and Table 2). Sink conditions were assured all over the assay, as shown for the mean value of *A*_*total*_ (Table 2), and even confirmed for all individual cumulative concentrations obtained for all tissues. Indeed, the maximum experimental concentration achieved in the receptor compartment was 24.9 µg/mL, far below 148.5 µg/mL which corresponds to 30% and 49.5 µg/mL (10%) of the maximum solubility of clotrimazole in the receptor solution (495 µg/mL) (Supplementary Materials, Table S3). These results further support tissue integrity maintenance throughout the 48 h assay, since the flux between the 26 and 48 h timeframe was lower than the one obtained between 0 and 24 h (Table 2).

### 3.5. Mass Balance

The Draft Guideline suggests that extraction of the donor chamber should be discriminative of the content recovered from the donor chamber, i.e., differentiate between the amount extracted from the tissue and the amount extracted from the donor chamber [3]. In this model, this distinction may prove difficult due to the formulation present on top of the tissue and retained in the tissue itself. Limitations on the mass balance results were detected. The mean mass balance value obtained for all products was between 80 and 82%, deviating from the ideal 90–110% established in the Draft Guideline (individual results obtained for all tissues are presented in Supplementary Materials Table S6). This could be due to the low amounts of drug that were extracted in each compartment, which may be highly affected even by small drug amount losses during the process of extraction. 

As previously discussed, the results obtained for insert #10 of assay#3 and for insert #1 of assay#5 were not used for the overall analysis of the study, since it was not possible to calculate the mass balance.

Concerning the receptor chamber, the 12-well plate washing started to be performed after assay#3 with 2 mL of methanol. This mass balance step was introduced in the bioequivalence studies to increase recovery of the drug in the event of possible adherence to the well plate material. Even with this washing step, no upgrade in the mass balance percentages was observed. The possibility of some of the drug being retained in the collagen support ring was also discarded by extracting this external support with 2 mL of methanol and no quantification was observed (data not shown). 

The differential recovery of the compartments may possibly be related to an increased loss of the drug, since the absolute amounts of drug are very low. A possible solution for this limitation may be the pooled extraction of all compartments (insert and tissue) in the same extraction cup, avoiding sample manipulation and, consequently, API loss. This was not tested because it did not comply with the Draft Guideline requirements.

Because all of the other validation parameters were in accordance with the requirement criteria, it was considered that this deviation in mass balance range, which occurred for all formulations, did not affect the comparison purposes of the bioequivalence study.

### 3.6. Bioequivalence Statistical Analysis

The statistical analysis results are presented in Table 5.

A 24 h timeframe is used more often in bioequivalence testing [3]. However, completing the study for up to 48 h was important to evidence more complete permeation profiles, even if it may be associated with higher risks of membrane damage. 

In all statistical comparisons, using either a 24 or 48 h timeframe, the test and comparator products (i.e., A and B) were within the equivalence interval of 80.00–125.00% range. On the other hand, both the negative control formulations (i.e., C and D) revealed inequivalence by being outside that same interval. These results validate the discriminatory power of the method for three different API concentrations (one lower and one higher than the test/comparator concentration) in topical formulations.

## 4. Discussion

The receptor solution selection and the experimental setup optimization that takes part in the pilot study enabled the distinction among 100% strength products (i.e., test and comparator) and 200% strength API (i.e., negative control). According to the EMA’s Draft Guideline, every bioequivalence study must be validated. In in vitro permeation studies, the validation process is based on concurrent results obtained with a negative control (with different API concentrations). This strategy assessed the method’s ability to discriminate different API concentrations in the same base. As previously mentioned, a negative control was already set in the pilot study to verify that sink conditions were gathered at all concentrations studied. An additional product with 50% API strength was also studied to better investigate the method’s discriminatory power through a wider API concentration range. 

The results obtained in the pilot study supported the discriminatory power of the method and adequacy of the defined experimental conditions for the bioequivalence study. Four products were statistically evaluated with *n* = 12 RhE tissue inserts each with an origin from five different batches.

The method complied with all validation parameters described in the Draft Guideline. Furthermore, low variability in permeation fluxes was obtained for all products tested. The first 24 h sample points were considered since higher flux rates were obtained in that timeframe. Equivalence between the test and comparator product was established. Both 50% and 200% demonstrated to be nonequivalent, supporting the method’s discriminatory power quality.

These results support the interest in RhE for bioequivalence studies and are in accordance with the literature. Schäfer-Korting et al. [28] used OECD reference substances with different solubilities (testosterone for lipophilic substances and caffeine for hydrophilic substances) and concluded that, although different commercially available RhE models overestimate those chemicals permeation, it was possible to establish the same ranking correlation between them (human and animal excised skin). Moreover, the RhE models demonstrated smaller and lower variable lag times [28]. To date, a prediction model for human skin uptake obtained from in vitro percutaneous absorption experiments with RhE, in agreement with the stepwise approach for validation studies of non-animal experiments described by ECVAM, is not yet available [60]. Unlike screening permeation experiments, bioequivalence studies are designed to compare two products in the same conditions, accounting for the variability of biological study systems. For such purposes, absolute permeation values and the skin uptake amount are not relevant, being only the maximum fluxes and the cumulative amount permeated statistically compared in accordance with the requirements of Draft Guideline on Quality and Equivalence of Topical Products CHMP/QWP/708282/2018 [3].

Tissue integrity was assessed by two methods in selected inserts. Firstly, there was the objective to understand the baseline values and report the tissue integrity during the assay. In fact, it is reasonable to expect that RhE models without maintenance medium lose their integrity over time, since they are living tissues. This was consistent with both TEER and TEWL findings. Although this could be seen as a drawback, results from the cumulative amount of clotrimazole did not greatly increase over time. In fact, the flux (J) until 24 h was always higher than the flux for the 26–48 h timeframe (Table 2 and Figure 3). This plateau-like curve (Figure 4) ensured the tissue’s ability to interact with the product, without tissue integrity issues. Otherwise, the amount of API considerably increased in the receptor solution for no apparent reason (since there was drug available in the donor compartment to permeate the skin). Sink conditions were kept throughout the study.

To assess the mass balance, the extraction of API from the product is a requirement, preferably performed within the pilot study. This parameter was evaluated and the recovery of clotrimazole from the product met the specified range determined by EMA (90–110%) [3] with a recovery of 103%. The main problem encountered in this step was that the mean mass balance recovery was approximately 81%, diverging to the left side interval considered by the EMA’s Draft Guideline (90–110%). Since we are working with very low concentrations of API, any variation in each extraction compartment may lead to small API losses that represent high percentages from the total. To overcome this limitation, it could be acceptable to gather some of the mandatory extractions, i.e., tissue and donor chamber extractions should be extracted together just for these bioequivalence studies that only compare permeation profiles, not the absorbed amount in the skin which will be assessed by stratum corneum tape stripping (Annex III from the EMA Draft Guideline [3]).

Clotrimazole, like many other molecules, is considered to experience high variability results and limited diffusion. The challenging problem to study and obtain a permeation profile with ex vivo human skin can be overcome using the advantageous higher permeation characteristic of RhE models when compared with human skin. On the other hand, it is expected that whenever the test substances are known to easily permeate the skin, this model may not be adequate for this purpose and excised human skin must be considered.

While promising, this modified method also presented several limitations: The skin model may lose viability throughout the assay, since it is not kept in culture medium and this fact can affect its integrity. In addition, the temperature of the assay used in this study did not correspond to the skin’s temperature (32 °C). It was modified to 37 °C, which is the temperature used in safety in vitro skin tests based on the same models, currently accepted by regulatory authorities (e.g., OECD TG 431, OECD TG439, and OECD TG498). There is the possibility that changes in temperature may change the properties of skin structures (such as lipids), which may affect skin permeation. Furthermore, the fact that sampling occurred at room temperature may somehow impact the results. However, the impact of this deviation is mitigated by the fact that the products (test and reference) had similar compositions and were compared under the same conditions throughout the study. 

## 5. Conclusions

This study showed that the human reconstructed epidermis is a valid model to be used as an alternative to ex vivo human skin permeation membrane for in vitro bioequivalence studies for clotrimazole, in accordance with the requirements of the Draft Guideline on Quality and Equivalence of Topical Products. This model has particular interest for IVPT studies involving drugs with limited skin permeability (that often present challenges for quantification) and also for highly variable drugs. Other future outcomes should be accomplished such as comparative results with other validated RhE models and further studies upon formulations containing APIs with different chemical characteristics, whether they are highly variable drugs or not. In addition, future work is needed to compare the results obtained at 32 °C with the results obtained at 37 °C.

## Figures and Tables

**Table 1:** Assay conditions used for the pilot study and for bioequivalence in vitro permeation testing.

	Pilot Study	Bioequivalence In Vitro Testing
**Sampling timepoints** (±2 min)	IQC, 3, 6, 12, 18, 21, 24, 42, 45, and 48 h (*n* = 9)	IQC, 2, 6, 12, 16, 18, 20, 22, 24, 26, 28, 30, 40, 42, 44, 46, and 48 h (*n* = 16)
**Total time**	48 h	
**Number of assays**	1 assay with 9 tissues: 3 tissues with comparator formulation 3 tissues with test formulation 1 tissue with negative control 20 mg/g formulation 1 tissue with placebo formulation 1 tissue as blank (no sample application)	5 assays representing 5 batches of RhE tissues totaling 12 tissues for each formulation tested
**Receptor solution**	PBS pH 7.4: propan-1,2-diol (60:40, *v*/*v*) with 0.02% sodium azide, 2 mL	
**Assay conditions**	37 °C (±1 °C), 5% CO_2_, ≥90% relative humidity	
**Agitation**	250 rpm	
**Applied formulation**	~15 mg	
**Sampling volume**	200 µL with receptor solution reposition (thermostatized at the temperature of the assay)	
**Membrane**	EPSKIN^®^ large/reconstructed human epidermis (area: 1.07 cm^2^)	
**Mass balance**		
Donor chamber extraction	5 mL methanol + 15 min of sonication + filtration PTFE 0.45 µm	5 mL methanol + 20 glass beads + 15 min of sonication + filtration PTFE 0.45 µm
Membrane extraction	2 mL methanol + 15 min of sonication + filtration PTFE 0.45 µm	2 mL methanol + 10 glass beads + 15 min of sonication + filtration PTFE 0.45 µm
Plate wash	Not performed	2 mL of methanol

**Table 2:** *A*_*total*_, J (for long timeframes) and J_max_ expressed as mean values and coefficients of variations (*n* = 12) for different timeframes (A—clotrimazole 10 mg/g cream; B—Canesten Antifungal Cream 10 mg/g cream; C—clotrimazole 20 mg/g cream; D—clotrimazole 5 mg/g cream).

	A	B	C	D
Flux, J, µg/cm^2^/h (coefficient of variation%)				
0–48 h	0.67 (17.68)	0.62 (17.80)	1.33 (10.95)	0.35 (10.95)
0–24 h	0.90 (20.73)	0.81 (15.20)	1.68 (15.99)	0.46 (11.69)
26–48 h	0.53 (12.42)	0.48 (22.55)	1.08 (12.57)	0.28 (23.62)
Cumulative amount, *A*_*total*_, µg/cm^2^ (coefficient of variation%)				
24 h	20.23 (20.68)	18.50 (15.72)	37.27 (16.50)	10.68 (12.83)
48 h	33.31 (17.46)	31.38 (17.46)	63.79 (13.79)	17.89 (11.13)
Maximum Flux, J_max_, µg/cm^2^/h (coefficient of variation%)				
	0.94 (19.65)	0.88 (13.97)	1.63 (18.15)	0.51 (21.43)

**Table 3:** TEER (Ωcm^2^) measurements performed on the day of reception of the tissues (D0), the day of sample application (D1), and 48 h after sample application (D3) for assay#3 and assay#4 using receptor solution, PBS pH 7.4 or maintenance medium at the top and at the bottom of the inserts to perform the measurements.

	Assay#3			Assay#4			
	Receptor Solution			PBS pH 7.4		Maintenance Medium	
Assay day	D0	D1	D3	D0	D3	D0	D3
Insert 13—Placebo formulation	11,518	1206	796	8832	525	8561	518
Insert 11—Comparator formulation	-	-	927	-	-	-	-

**Table 4:** TEWL (g/m^2^ h) measurements performed before sample application (D1) and after the assay ended (D3).

	Assay#1	Assay#2		Assay#3		Assay#4	Assay#5
Insert #	12	13	12	13	11	8	4
Day D1							
Mean (*n*)	28.0 (4)	15.4 (4)	-	27.6 (9)	-	25.5 (6)	27.9 (9)
SD	1.9	1.9	-	1.7	-	1.6	1.1
CV%	6.8	12.2	-	6.3	-	6.3	4.1
Day D3							
Mean (*n*)	49.2 (4)	35.0 (9)	22.3 (9)	28.8 (9)	32.7 (8)	38.1 (8)	46.5 (8)
SD	5.2	0.6	1.6	3.0	4.3	1.3	0.9
CV%	10.6	1.8	7.0	10.5	13.1	3.5	1.9

**Table 5:** Statistical analysis for *A*_*total*_ results at 24 and 48 h, as well as J_max_ between the test and comparator formulations (A and B) and between the test formulations (A, C, and D) themselves. (A—test product; B—comparator product; C—“negative control” with 200% strength of the test formulation; D—“negative control” with 50% strength of the test formulation). Results within the 80.00–125.00% range indicate bioequivalence.

Cumulative Amount at 24 h (*A*_*total*_)				
	Test	A	C	D
Comparator				
A		-	173.63–195.91	41.35–69.62
B		97.46–119.73	189.97–214.33	45.24–69.61
**Cumulative Amount at 48 h (*A*_*total*_)**				
	Test	A	C	D
Comparator				
A		-	181.68–202.60	42.90–63.82
B		94.80–116.95	192.35–214.50	45.42–67.56
**Maximum Flux (J_max_)**				
	Test	A	C	D
Comparator				
A		-	162.22–186.31	42.62–66.71
B		93.57–119.19	172.57–198.19	42.34–70.96

## Data Availability

Not applicable.

## Supplementary Materials

The following supporting information can be downloaded at: https://www.mdpi.com/article/10.3390/pharmaceutics14081554/s1, Figure S1: Schematic representation of the experimental setup process from sample application to analysis of the results; Table S1: Products under test for bioequivalence study and IVPT method validation; Table S2: Information and justifications of all deviations from the IVPT standard testing according the Draft Guideline EMA/CHMP/QWP/708282/2018 throughout the study design performed in the manuscript; Table S3: Solubility studies for clotrimazole were carried out by shaking an excess amount of drug with the solutions described. The samples were incubated at 37 °C for 24 h. After the incubation time, the samples were centrifuged (10,000 rpm, 10 min). The supernatants were collected and filtered through 0.22 µm and injected into HPLC for analysis; Table S4: Study design performed assigning each EPISKIN^®^ insert and EPISKIN^®^ batch to a drug product (codification from A—test product; B—comparator product; C—“negative control” with 200% strength of test formulation; D—“negative control” with 50% strength of test formulation); Table S5: TEWL defined parameters; Table S6: Mass balance results by summing the amount of clotrimazole in the donor chamber (removed from the top of the tissue after 48 h assay), retained in the tissue (extracted from the tissue), in the plate wash (extracted from each well of the six-well plate), and the cumulative amount in the receptor chamber.

## References

[1] FDA (2016). Draft Guidance on Acyclovir.

[2] (2014). Guideline on Quality of Transdermal Patches.

[3] (2018). Draft Guideline on Quality and Equivalence of Topical Products.

[4] OECD (2004). Guidance Document for the Conduct of Skin Absorption Studies.

[5] WHO (2006). Multisource (Generic) Pharmaceutical Products: Guidelines on Registration Requirements to Establish Interchangeability.

[6] Kassis V., Sondergaard J. (1982). Heat-separation of normal human skin for epidermal and dermal prostaglandin analysis. Arch. Dermatol. Res..

[7] Frank J.D., Manson J.M., Cartwright M.E. (1995). Separation of epidermis from dermis in the Rhesus monkey. Exp. Dermatol..

[8] Reichling J., Landvatter U., Wagner H., Kostka K.-H., Schaefer U.F. (2006). In vitro studies on release and human skin permeation of Australian tea tree oil (TTO) from topical formulations. Eur. J. Pharm. Biopharm..

[9] Pantelić I., Ilić T., Marković B., Savić S., Lukić M., Savić S. (2018). A stepwise protocol for drug permeation assessment that combines heat-separated porcine ear epidermis and vertical diffusion cells. Hem. Ind..

[10] Franz T., Lehman P.A., Raney S. (2009). Use of excised human skin to assess the bioequivalence of topical products. Skin Pharmacol. Physiol..

[11] Lehman P.A., Franz T.J. (2014). Assessing Topical Bioavailability and Bioequivalence: A Comparison of the In vitro Permeation Test and the Vasoconstrictor Assay. Pharm. Res..

[12] Brain K.R., Chilcott R.P., Chilcott R.P., Price S. (2008). Physicochemical Factors Affecting Skin Absorption.

[13] Schäfer-Korting M., Bock U., Diembeck W., Düsing H.-J., Gamer A., Haltner-Ukomadu E., Hoffmann C., Kaca M., Kamp H., Kersen S. (2008). The Use of Reconstructed Human Epidermis for Skin Absorption Testing: Results of the Validation Study. Altern. Lab. Anim..

[14] Haberland A., Schreiber S., Maia C.S., Rübbelke M., Schaller M., Korting H., Kleuser B., Schimke I., Schäfer-Korting M. (2006). The impact of skin viability on drug metabolism and permeation—BSA toxicity on primary keratinocytes. Toxicol. In Vitro.

[15] Kandárová H., Liebsch M., Genschow E., Gerner I., Traue D., Slawik B., Spielmann H. (2004). Optimisation of the EpiDerm test protocol for the upcoming ECVAM validation study on in vitro skin irritation tests. ALTEX.

[16] DB-ALM Reconstructed Skin Models for Percutaneous Absorption Testing—Summary (Percutaneous Absorption). Method Summary 2010, 1–8. https://jeodpp.jrc.ec.europa.eu/ftp/jrc-opendata/EURL-ECVAM/datasets/DBALM/LATEST/online/dbalm.html.

[17] Borgia S.L., Schlupp P., Mehnert W., Schäfer-Korting M. (2008). In vitro skin absorption and drug release—A comparison of six commercial prednicarbate preparations for topical use. Eur. J. Pharm. Biopharm..

[18] Hu T., Bailey R.E., Morrall S.W., Aardema M.J., Stanley L.A., Skare J.A. (2009). Dermal penetration and metabolism of p-aminophenol and p-phenylenediamine: Application of the EpiDerm™ human reconstructed epidermis model. Toxicol. Lett..

[19] Sugibayashi K., Hayashi T., Matsumoto K., Hasegawa T. (2004). Utility of a Three-Dimensional Cultured Human Skin Model as a Tool to Evaluate the Simultaneous Diffusion and Metabolism of Ethyl Nicotinate in Skin. Drug Metab. Pharmacokinet..

[20] Monti D., Brini I., Tampucci S., Chetoni P., Burgalassi S., Paganuzzi D., Ghirardini A. (2008). Skin Permeation and Distribution of Two Sunscreens: A Comparison between Reconstituted Human Skin and Hairless Rat Skin. Skin Pharmacol. Physiol..

[21] Mavon A., Raufast V., Redoulès D. (2004). Skin absorption and metabolism of a new vitamin E prodrug, delta-tocopherol-glucoside: In vitro evaluation in human skin models. J. Control. Release.

[22] Ackermann K., Borgia S.L., Korting H.C., Mewes K.R., Schäfer-Korting M. (2010). The Phenion^®^ Full-Thickness Skin Model for Percutaneous Absorption Testing. Skin Pharmacol. Physiol..

[23] Fleischli F., Morf F., Adlhart C. (2015). Skin concentrations of topically applied substances in reconstructed human epidermis (RHE) compared with human skin using in vivo confocal Raman microscopy. Chimia.

[24] Schmook F.P., Meingassner J.G., Billich A. (2001). Comparison of human skin or epidermis models with human and animal skin in in-vitro percutaneous absorption. Int. J. Pharm..

[25] Gabbanini S., Lucchi E., Carli M., Berlini E., Minghetti A., Valgimigli L. (2009). In vitro evaluation of the permeation through reconstructed human epidermis of essentials oils from cosmetic formulations. J. Pharm. Biomed. Anal..

[26] Lent E.M., Maistros K.J., Oyler J.M. (2020). In vitro dermal absorption of carfentanil. Toxicol. In Vitro.

[27] Godin B., Touitou E. (2007). Transdermal skin delivery: Predictions for humans from in vivo, ex vivo and animal models. Adv. Drug Deliv. Rev..

[28] Schäfer-Korting M., Bock U., Gamer A., Haberland A., Haltner-Ukomadu E., Kaca M., Kamp H., Kietzmann M., Korting H.C., Krächter H.-U. (2006). Reconstructed Human Epidermis for Skin Absorption Testing: Results of the German Prevalidation Study. Altern. Lab. Anim..

[29] Supe S., Takudage P. (2020). Methods for evaluating penetration of drug into the skin: A review. Skin Res. Technol..

[30] Alves T., Arranca D., Martins A., Ribeiro H., Raposo S., Marto J. (2021). Complying with the Guideline for Quality and Equivalence for Topical Semisolid Products: The Case of Clotrimazole Cream. Pharmaceutics.

[31] Bolla P.K., Meraz C.A., Rodriguez V.A., Deaguero I., Singh M., Yellepeddi V.K., Renukuntla J. (2019). Clotrimazole Loaded Ufosomes for Topical Delivery: Formulation Development and In-Vitro Studies. Molecules.

[32] Taboada J., Grooters A.M., Maddison J.E., Page S.W., Church D.B. (2008). Systemic antifungal therapy. Small Animal Clinical Pharmacology.

[33] Waugh C.D., Enna S.J., Bylund D.B. (2007). Clotrimazole. xPharm: The Comprehensive Pharmacology Reference.

[34] Stamatialis D., Rolevink H.H.M., Koops G.H. (2002). Controlled transport of timolol maleate through artificial membranes under passive and iontophoretic conditions. J. Control. Release.

[35] Swarbrickx J., Lee G., Brom J., Gensmantel N.P. (1984). Drug Permeation Through Human Skin II: Permeability of Ionizable Compounds. J. Pharm. Sci..

[36] Shanmugam S., Song C.-K., Nagayya-Sriraman S., Baskaran R., Yong C.-S., Choi H.-G., Kim D.-D., Woo J.S., Yoo B.-K. (2009). Physicochemical characterization and skin permeation of liposome formulations containing clindamycin phosphate. Arch. Pharm. Res..

[37] der Yu C., Higuchi W.I., Ho N.F., Fox J.L., Flynn G.L. (1980). Physical model evaluation of topical prodrug delivery—Simultaneous transport and bioconversion of vidarabine-5′-valerate III: Permeability differences of vidarabine and n-pentanol in components of hairless mouse skin. J. Pharm. Sci..

[38] Wang T., Kasichayanula S., Gu X. (2006). In vitro permeation of repellent DEET and sunscreen oxybenzone across three artificial membranes. Int. J. Pharm..

[39] Trommer H., Neubert R.H. (2006). Overcoming the stratum corneum: The modulation of skin penetration. A review. Skin Pharmacol. Physiol..

[40] Carrer V., Alonso C., Pont M., Zanuy M., Cordoba M., Espinosa S., Barba C., Oliver M.A., Marti M., Coderch L. (2020). Effect of propylene glycol on the skin penetration of drugs. Arch Dermatol. Res..

[41] Patel A., Iliopoulos F., Caspers P., Puppels G., Lane M. (2021). In Vitro–In Vivo Correlation in Dermal Delivery: The Role of Excipients. Pharmaceutics.

[42] Roberts M.S., Cheruvu H.S., Mangion S.E., Alinaghi A., Benson H.A., Mohammed Y., Holmes A., van der Hoek J., Pastore M., Grice J.E. (2021). Topical drug delivery: History, percutaneous absorption, and product development. Adv. Drug Deliv. Rev..

[43] ICH (2005). Validation of Analytical Procedures: Text and Methodology Q2(R1).

[44] CDER (1994). Reviewer Guidance: Validation of Chromatographic Methods.

[45] Abdayem R., Callejon S., Portes P., Kirilov P., Demarne F., Pirot F., Jannin V., Haftek M. (2015). Modulation of transepithelial electric resistance (TEER) in reconstructed human epidermis by excipients known to permeate intestinal tight junctions. Exp. Dermatol..

[46] Alexander F.A., Wiest J. (2016). Automated transepithelial electrical resistance measurements of the EpiDerm reconstructed human epidermis model. Annu. Int. Conf. IEEE Eng. Med. Biol. Soc..

[47] Chacón M., Vázquez N., Persinal-Medina M., Alonso-Alonso S., Pevida M., Llames S., Baamonde B., Quiros L., Merayo-Lloves J., Meana Á. (2020). Development of an in-house reconstructed human epidermis model as an alternative method in skin corrosion assessment. Toxicol. In Vitro.

[48] Wei Z., Liu X., Ooka M., Zhang L., Song M.J., Huang R., Kleinstreuer N.C., Simeonov A., Xia M., Ferrer M. (2020). Two-Dimensional Cellular and Three-Dimensional Bio-Printed Skin Models to Screen Topical-Use Compounds for Irritation Potential. Front. Bioeng. Biotechnol..

[49] Gomaa Y.A., Morrow D.I., Garland M.J., Donnelly R.F., El-Khordagui L.K., Meidan V.M. (2010). Effects of microneedle length, density, insertion time and multiple applications on human skin barrier function: Assessments by transepidermal water loss. Toxicol. In Vitro.

[50] Klang V., Haberfeld S., Hartl A., Valenta C. (2012). Effect of γ-cyclodextrin on the in vitro skin permeation of a steroidal drug from nanoemulsions: Impact of experimental setup. Int. J. Pharm..

[51] Berthaud F., Smith B., Boncheva M. (2013). The impact of surface loading and dosing scheme on the skin uptake of fragrances. Toxicol. Vitr..

[52] Netzlaff F., Kostka K.-H., Lehr C.-M., Schaefer U.F. (2006). TEWL measurements as a routine method for evaluating the integrity of epidermis sheets in static Franz type diffusion cells in vitro. Limitations shown by transport data testing. Eur. J. Pharm. Biopharm..

[53] Elmahjoubi E., Frum Y., Eccleston G.M., Wilkinson S.C., Meidan V.M. (2009). Transepidermal water loss for probing full-thickness skin barrier function: Correlation with tritiated water flux, sensitivity to punctures and diverse surfactant exposures. Toxicol. In Vitro.

[54] Garcia N., Doucet O., Bayer M., Fouchard D., Zastrow L., Marty J.P. (2002). Characterization of the barrier function in a reconstituted human epidermis cultivated in chemically defined medium. Int. J. Cosmet. Sci..

[55] Dreher F., Fouchard F., Patouillet C., Andrian M., Simonnet J.T., Benech-Kieffer F. (2002). Comparison of cutaneous bioavailability of cosmetic preparations containing caffeine or alpha-tocopherol applied on human skin models or human skin ex vivo at finite doses. Skin Pharmacol. Appl. Skin Physiol..

[56] van der Bijl P., van Eyk A.D., Thompson I.O. (1998). Penetration of human vaginal and buccal mucosa by 4.4-kd and 12-kd fluorescein-isothiocyanate-labeled dextrans. Oral Surg. Oral Med. Oral Pathol. Oral Radiol. Endod..

[57] Shin S.H., Rantou E., Raney S.G., Ghosh P., Hassan H., Stinchcomb A. (2020). Cutaneous Pharmacokinetics of Acyclovir Cream 5% Products: Evaluating Bioequivalence with an In Vitro Permeation Test and an Adaptation of Scaled Average Bioequivalence. Pharm. Res..

[58] Shin S.H., Srivilai J., Ibrahim S.A., Strasinger C., Hammell D.C., Hassan H.E., Stinchcomb A. (2018). The Sensitivity of In Vitro Permeation Tests to Chemical Penetration Enhancer Concentration Changes in Fentanyl Transdermal Delivery Systems. AAPS PharmSciTech.

[59] Oh L., Yi S., Zhang D., Shin S.H., Bashaw E. (2019). In Vitro Skin Permeation Methodology for Over-The-Counter Topical Dermatologic Products. Ther. Innov. Regul. Sci..

[60] Hartung T., Bremer S., Casati S., Coecke S., Corvi R., Fortaner S., Gribaldo L., Halder M., Hoffmann S., Roi A.J. (2004). A Modular Approach to the ECVAM Principles on Test Validity. Altern. Lab. Anim..

