# Supplementary material for: Reconstructed Human Epidermis: An Alternative Approach for In Vitro Bioequivalence Testing of Topical Products
Source: Pharmaceutics. 2022 Jul 26;14(8):1554. doi: 10.3390/pharmaceutics14081554 (PMC9331624; doi:10.3390/pharmaceutics14081554)
Supplement: Supplementary file 1 [file pharmaceutics-14-01554-s001.zip › pharmaceutics-1612945-supplementary.pdf]

SUPPLEMENTARY MATERIAL

Figure S1. Schematic representation of the experimental setup process from sample application to analysis of the results.

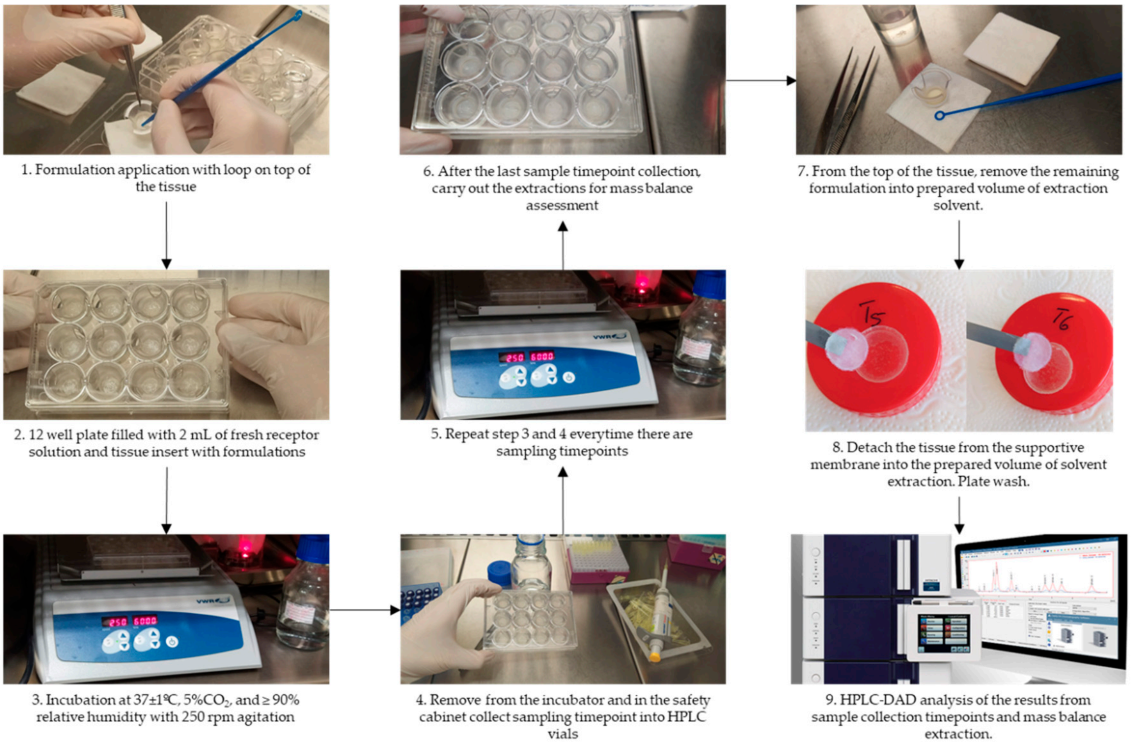

Table S1. Products under test for bioequivalence study and IVPT method validation.

**Product**	**Comparator product**	**Test product**	**Negative controls**	
**Code**	B	A	C	D
**Name**	Canesten Antifungal Cream 10 mg/g cream	Clotrimazole 10 mg/g cream	Clotrimazole 20 mg/g cream	Clotrimazole 5 mg/g cream
**Batch number**	BXPJREH	0944G	D2052	D1796
**Strength**	10 mg/g	10 mg/g	20 mg/g	5 mg/g
**Dosage form**	Cream			
**API**	Clotrimazole			
**Marketing Authorization Holder**	Bayer®	Basi®		
**Excipients**	Sorbitan stearate, polysorbate 60, cetyl palmitate, ceterayl alcohol, octyldodecanol, benzyl alcohol, and purified water	Cetyl palmitate, octyldodecanol, polysorbate 60, sorbitan stearate, benzyl alcohol, cetostearyl alcohol, and purified water		

Table S2. Information and justifications on experimental decisions including all deviations from the IVPT standard testing according to Draft Guideline EMA/CHMP/QWP/708282/2018 throughout this study design

	**Draft Guideline on quality and equivalence of topical products EMA/CHMP/QWP/708282/2018**	**Modified bioequivalence IVPT method performed**
Skin membrane	"It is recommended to use ex vivo adult human skin"; "Different skin preparations can be used"	This is the main alteration between the recommendation presented in the Draft Guideline and the modified method developed. Because of the high variability encountered in ex vivo human skin, the RhE model was defined for bioequivalence in vitro testing
	"The skin integrity should be checked prior and after each experiment"	The skin integrity was checked by two methods: TEWL and TEER (for more information see section 2.6.5.)
	"Skin from different donor should be chosen"<br>"The number of skin donors should not be less than 12, with at least 2 replicates per donor"	RhE model cannot be seen as a model that could be segregated by donor. Instead, the cells that are grown in the insert are from a pool with several donors. In order to fulfill this condition in the modified IVPT model, at least 12 RhE inserts were performed for each formulation originated from at least 5 different batches accounting for inter and intra batch variability Please refer to further discussion in Section 2.2.
	"The apparatus should ensure consistent temperature control throughout the duration of the experiment. The skin surface temperature should be stable at 32±1°C."	The RhE insert plates are placed in an incubator during the timeframe of the study. The incubator must be able to be stable at 37±1ºC with 5% CO2 and >90% relative humidity. These incubation conditions were performed to maintain the RhE inserts in their optimum maintenance environment as suggested for validated safety tests with this model.
Receptor medium	"Sink conditions should be confirmed as described with IVRT (Annex 1)."	Sink conditions were always checked and the API concentration in the receptor solution did not surpass 30% of the total amount applied on the top of the insert (see section 2.6.2.)
	"The receptor medium should be aqueous buffer, unless otherwise justified. Evidence should be provided that the chosen receptor medium does not compromise the skin barrier integrity throughout the test."	The receptor solution is constituted by an aqueous buffer at pH 7.4 and Propan-1,2-diol in the proportion of 60:40% V/V, respectively. For more support about this receptor solution see section 2.6.2.<br>Skin integrity was measured by TEER and TEWL prior and after the modified in vitro permeation testing (section 2.6.5)
	"The inclusion of an anti-microbial agent in the receptor medium, to mitigate potential bacterial decomposition of the skin membrane, is acceptable, but it should not interfere with the properties of the skin or the assay."	Sodium azide was used in a 0.02% (w/V) concentration in the receptor solution (see section 2.6.2.). Absence of interference was assessed through integrity measurements.
	"The number of sampling time points should be sufficient to obtain meaningful profiles…"	16 sampling time points were used in order to construct a meaningful profile (Table 1).
	"The recommended dosing amount should be in the rage of 2-15mg/cm2, based on SmPC posology, unless otherwise justified."	It was established as 15 mg/cm2. For more information refer to section 2.6.1.
	"To identify potential contamination and/or interferences, pre-dose samples collected from each diffusion cell and a parallel non-dosed blank control skin experiment are recommended"	IQC (Internal Quality Control) was performed prior to initiating the bioequivalence in vitro testing (Table 1)<br>A diffusion cell was not used in thisstudy. Instead the RhE inserts were directly placed in a 12-well plate which was maintained in continuous agitation.
	"For low strength drug product, the analytical methods should be sensitive enough to quantify the amount of drug in the receptor solution at various time points and be appropriately validated"	Quantifiable API was assured from the first sampling timepoint for the lowest API concentration formulation. The method was validated according to ICH guidelines.

Table S3. Solubility studies for clotrimazole were carried out by shaking an excess amount of drug with the solutions described. The samples were incubated at 37ºC for 24 hours. After incubation time, the samples were centrifuged (10 000 rpm, 10 minutes). The supernatants were collected and filtered through 0.22 μm and injected into HPLC for analysis.

Solubility solutions tested	Concentration (ug/mL)
PBS pH 7.4	0.65
PBS pH 7.4 : Ethanol (75:25, %V/V)	4.3
PBS pH 7.4 : Propylene glycol (60:40, %V/V)	495.0
PBS pH 7.4 : Polyethylene glycol 400 : (60:40, %V/V)	764.8
PBS pH 7.4 : Poloxamer (0.5 %m/V)	4.8
PBS pH 7.4 : Poloxamer (1 %m/V)	26.8
MeOH	816.9

Table S4. Study design performed assigning each EPISKIN® insert and EPISKIN® batch to a drug product (codification from A – test product; B – comparator product; C – "negative control" with 200% strength of test formulation; D- "negative control" with 50% strength of test formulation).

	EPISKIN® Insert#											
**Assay# and EPISKIN® Batch**	1	2	3	4	5	6	7	8	9	10	11	12
Assay#1 Batch 20 EPIS 034	A	A	A	B	B	B	C	C	C	D	D	D
Assay#2 Batch 20 EPIS 036	C	C	C	A	A	A	B	B	B	D	D	D
Assay#3 Batch 20 EPIS 041	D	D	D	C	C	C	A	A	A	B	B	B
Assay#4 Batch 20 EPIS 043	B	B	D	D	A	A	C	C	-	-	-	-
Assay#5 Batch 20 EPIS 044	A	B	C	B	A	D	-	-	-	-	-	-

Table S5. TEWL defined parameters.

**Cap factor**	**Time (seconds)**		**Moving average interval (seconds)**	**Target precision (StDev, g/m^2g)**
	Minimum	Maximum		
0.5	60	300	30	0.075

Table S6. Mass balance results by summing the amount of clotrimazole in the donor chamber (removed from the top of the tissue after 48-hour assay), in the tissue (extracted from the tissue), in plate wash (extracted from each well of the 6-well plate) and the cumulative amount in the receptor chamber.

**Formulation A**	**Assay #1**			**#2**			**#3**			**#4**		**#5**	**Mean**	**SD**	**%CV**
Mass Balance (%)	66	85	78	81	61	98	69	89	99	76	77	81	80.0	11.7	14.6
Donor Chamber (μg)	54.3	79.4	57.1	66.7	60.3	100.9	74.7	72	87.2	101.3	101.3	87.4	78.6	17.2	21.9
Tissue (μg)	8.1	24.6	11	21.4	11.3	28.9	21.3	47.8	53.5	6.4	8.8	3.5	20.6	16.2	78.7
Atotal 48h (μg)	26.3	31.9	32.4	31.9	31.2	39.1	43.1	45	45.8	33.3	36.1	31.6	35.6	6.2	17.5
Plate wash (μg)	-	-	-	-	-	-	0.4	0.3	0.3	0.4	0.1	0.2	0.3	0.1	41.3
**Formulation B**	**Assay #1**			**#2**			**#3**			**#4**		**#5**	**Mean**	**SD**	**%CV**
Mass Balance (%)	89	87	81	63	70	83	81	75	96	79	86	80	80.8	8.7	10.8
Donor Chamber (μg)	70.3	72.6	73.1	59.4	60.2	78.7	59.4	33.8	101.3	101.3	88.4	49.3	70.7	20.1	28.5
Tissue (μg)	17.7	13.8	19	12.4	8.1	10.8	14.1	19.4	20.8	4.8	17.6	4.9	13.6	5.6	40.9
Atotal 48h (μg)	36.1	31.5	29.8	29.6	26.8	32.1	34	31.6	45.8	26.9	37.4	42.5	33.7	5.9	17.5
Plate wash (μg)	-	-	-	-	-	-	0.4	0.3	0.6	0.4	0.5	0.3	0.4	0.1	28.1
**Formulation C**	**Assay #1**			**#2**			**#3**			**#4**		**#5**	**Mean**	**SD**	**%CV**
Mass Balance (%)	90	90	84	79	78	78	85	88	93	80	85	57	82.3	9.4	11.5
Donor Chamber (μg)	242.7	199.1	140.3	127.9	141	126.3	124.2	119.3	173.7	101.3	101.3	99.5	141.4	43.5	30.8
Tissue (μg)	4.0	14.6	39.8	47.5	38.7	40.5	56.2	35.1	82.5	13.2	30.4	36.4	36.6	20.8	57.0
Atotal 48h (μg)	70.3	69.2	64.8	62.2	56.4	53.4	83.1	74	80.9	75.3	70.8	58.7	68.3	9.4	13.8
Plate wash (μg)	-	-	-	-	-	-	0.5	0.5	0.6	0.9	0.7	0.4	0.6	0.2	29.8
**Formulation D**	**Assay #1**			**#2**			**#3**			**#4**		**#5**	**Mean**	**SD**	**%CV**
Mass Balance (%)	90	82	68	88	80	101	76	83	76	73	83	69	80.8	9.4	11.6
Donor Chamber (μg)	59.4	34.7	25.7	41.4	28.5	41.4	27.6	34.7	26.8	101.3	101.3	33.5	46.4	27.3	58.8
Tissue (μg)	4.2	11.8	7.8	8	10.4	13.8	7.7	20.8	10.1	11.5	5.4	4.2	9.6	4.6	48.2
Atotal 48h (μg)	17.2	21.7	17.1	19	19.1	20.5	16.9	21.6	17.6	19	23.1	16.9	19.1	2.1	11.2
Plate wash (μg)	-	-	-	-	-	-	0.1	0.1	0.1	0.2	0.2	0.1	0.1	0.1	38.7
